# The Role of Vitamin B Complex in Periodontal Disease: A Systematic Review Examining Supplementation Outcomes, Age Differences in Children and Adults, and Aesthetic Changes

**DOI:** 10.3390/nu17071166

**Published:** 2025-03-27

**Authors:** Roxana Buzatu, Magda Mihaela Luca, Bogdan Andrei Bumbu

**Affiliations:** 1 Department of Dental Aesthetics, Faculty of Dental Medicine, “Victor Babes” University of Medicine and Pharmacy Timisoara, Revolutiei Boulevard 9, 300041 Timisoara, Romania; roxana.buzatu@umft.ro; 2 Pediatric Dentistry Research Center (Pedo-Research), Department of Pediatric Dentistry, Faculty of Dental Medicine, “Victor Babes” University of Medicine and Pharmacy Timisoara, Eftimie Murgu Square 2, 300041 Timisoara, Romania; 3 Department of Dental Medicine, Faculty of Medicine and Pharmacy, University of Oradea, 410073 Oradea, Romania; bogdanbumbu@uoradea.ro

**Keywords:** vitamin B complex, periodontal disease, folate, nutrition, oral health

## Abstract

**Background and Objectives:** Among nutritional factors implicated in periodontal health, the vitamin B complex—particularly folate (vitamin B9), cobalamin (B12), thiamine (B1), and riboflavin (B2)—has gained attention for its role in immunomodulation and tissue repair. This systematic review aims to synthesize current evidence on whether adequate vitamin B complex intake or status is associated with improved periodontal outcomes. **Methods:** A systematic search was performed in PubMed, Scopus, and Web of Science for observational studies investigating vitamin B complex intake or status in relation to periodontal disease indicators. Articles were screened according to PRISMA guidelines, and five studies met inclusion criteria. **Results:** Five observational studies were included. In older adults, each standard deviation increase in serum folate was associated with an approximate 26% reduction in periodontal disease odds ratio (OR = 0.74, 95% confidence interval (CI) 0.58–0.93). Among young adult women, inadequate riboflavin (B2) and pyridoxine (B6) intake correlated with higher community periodontal index (CPI) scores (*p* < 0.05). In a large NHANES-based cohort, insufficient thiamine (B1) intake yielded a 33% higher likelihood of severe periodontitis (*p* < 0.05), while adequate riboflavin was protective (OR = 0.90). Another dose–response analysis (n = 8959) indicated up to a 30% risk reduction for moderate folate or B1 intake, but no extra benefit with excessive intake. Finally, a UK Biobank analysis (n = 9476) showed that those in the highest quartile of a “high micronutrient” dietary pattern—including vitamins B6 and folate—had a 24% lower risk of self-reported periodontal disease (OR = 0.76, 95% CI 0.65–0.90) compared to the lowest quartile. **Conclusions:** Across diverse populations, inadequate vitamin B complex intake—especially folate—was consistently linked to worse periodontal outcomes.

## 1. Introduction

Periodontal disease is recognized as a major global health concern, affecting millions of people and imposing substantial clinical and economic burdens [1,2]. Characterized by chronic inflammation in the periodontium, periodontitis is known to cause progressive tissue destruction and alveolar bone loss, ultimately leading to tooth mobility and potential tooth loss [3,4]. In addition to its oral manifestations, evidence increasingly supports systemic ramifications, linking periodontitis with cardiovascular disease, diabetes, and other conditions through inflammation-mediated mechanisms [5,6,7,8].

The B vitamin complex comprises water-soluble vitamins that play essential roles in cell metabolism. Unlike fat-soluble vitamins that can be stored in the body’s fatty tissues, water-soluble B vitamins must be continually replenished as they are not retained for long periods. They are excreted through urine, necessitating consistent dietary intake to maintain adequate levels. These vitamins facilitate crucial processes such as energy production, enzyme function, and neurological health, making them vital for maintaining overall physiological and biochemical stability. Therefore, deficiencies in essential vitamins, particularly those in the vitamin B complex, can exacerbate the inflammatory processes at the core of periodontitis [9,10,11,12]. Vitamins like folate (B9) and cobalamin (B12) are critical for cellular functions such as DNA synthesis and neural function, while thiamine (B1) and riboflavin (B2) support energy metabolism, all vital for maintaining healthy periodontal tissue [13,14,15,16].

Over the past decade, several epidemiological and clinical studies have investigated whether deficiencies or suboptimal intake of these B vitamins exacerbate periodontal breakdown [8,17]. For instance, prospective data suggest that inadequate B vitamin status may impair wound healing, intensify alveolar bone resorption, or weaken the host response to microbial challenges [7,16]. Conversely, maintaining adequate B vitamin intake might bolster epithelial barrier function and mitigate oxidative stress, potentially reducing gingival inflammation and pocket depth [9,18]. Despite these mechanistic hypotheses, evidence remains partly inconclusive, owing to variability in how periodontal disease and dietary intake are measured [19,20].

The literature features heterogeneous methodological approaches. Some studies rely on self-reported dietary questionnaires, while others measure serum biomarkers such as folate or cobalamin [16]. Concurrently, periodontal assessments differ, ranging from full-mouth clinical attachment level (CAL) and probing depth (PD) measurements to partial-mouth protocols or self-reported signs such as bleeding gums or tooth looseness [3,8]. This heterogeneity underscores the complexity of synthesizing results into definitive recommendations. Nonetheless, recurring themes point to suboptimal B vitamin levels as a plausible risk factor for periodontal deterioration [11,12].

Evidence from robust datasets such as the National Health and Nutrition Examination Survey (NHANES) indicates that low folate levels may correlate with heightened odds of periodontitis among older adults [16]. Additional cross-sectional research links insufficient B1 or B2 with worse periodontal indices in broader adult populations [9]. Meanwhile, investigators continue to explore potential dose–response relationships, hypothesizing that up to a certain threshold, B vitamin supplementation confers protective effects on the periodontium [7,10]. Although the exact mechanism remains to be fully characterized, plausible biological pathways involve modulating homocysteine levels, aiding tissue repair, and reducing oxidative stress within gingival tissues [6]. Moreover, the vitamin B complex is integral to periodontal health due to its role in immunomodulation and the maintenance of mucosal integrity. Folate (B9) and riboflavin (B2), components of the vitamin B complex, are particularly significant in modulating inflammatory responses and sustaining epithelial tissues within the oral cavity. Adequate levels of these vitamins may confer a protective effect against periodontitis by enhancing host immune defenses and facilitating the repair of periodontal tissues [6,7,10].

Given these developments, a systematic synthesis focusing specifically on the vitamin B complex in periodontal disease is timely. While reviews exist on broader dietary patterns [13,15] or on single nutrients such as vitamin C and D [10,20], fewer publications highlight how combined B vitamins might work synergistically to modulate oral and systemic inflammation [14,16].

The hypothesis of the study is that adequate intake or status of the vitamin B complex, which includes folate (vitamin B9), cobalamin (B12), thiamine (B1), and riboflavin (B2), is associated with improved periodontal outcomes.

## 2. Materials and Methods

### 2.1. Eligibility Criteria and Information Sources

This review therefore addresses crucial questions: Is there a consistent association between vitamin B deficiencies and higher periodontitis risk, age differences, and aesthetic differences? Do certain B vitamins, like folate, exhibit a more pronounced effect? Are there threshold or dose–response relationships beyond which additional B vitamin intake confers minimal benefit? By examining these lines of inquiry, we aim to inform clinicians, researchers, and policymakers regarding the potential utility of incorporating vitamin B-focused dietary guidance into comprehensive periodontal disease management.

We adhered to the Preferred Reporting Items for Systematic Reviews and Meta-Analyses (PRISMA) guidelines to ensure methodological transparency, and registered our protocol in the Open Science Framework, with the registration code osf.io/sd5pg.

Inclusion criteria comprised as follows: (1) Studies must involve adult human participants aged 18 years or older; (2) studies must assess the status of the vitamin B complex—whether through dietary intake, supplementation, or serum biomarkers; (3) studies must measure periodontal disease using indicators such as clinical attachment loss (CAL), probing depth (PD), bleeding on probing, or validated self-reports; and (4) articles must be written in English. Exclusion criteria were as follows: (1) Reviews, case reports, and conference abstracts; (2) non-human or in vitro studies; (3) studies not measuring periodontal outcomes; and (4) duplicate studies.

We systematically searched PubMed, Scopus, and Web of Science. The search strategy combined terms such as “periodontal disease”, “periodontitis”, “gingivitis”, “vitamin B complex”, “folate”, “vitamin B12”, “thiamine”, “riboflavin”, “nutrition”, and “diet”, using Boolean operators. Articles in English were included, and references of retrieved articles were hand-searched for additional eligible studies. Two reviewers screened titles and abstracts independently, resolving discrepancies by discussion or, if necessary, by a third reviewer’s decision. Full texts of potentially relevant studies were then examined.

### 2.2. Search Strategy and Study Selection

To ensure a comprehensive and unbiased selection of articles, we developed a multi-step search strategy. Initially, we formulated a set of key terms: (“periodontitis” OR “periodontal disease” OR “gingivitis”) AND (“vitamin B complex” OR “vitamin B9” OR “folic acid” OR “folate” OR “vitamin B12” OR “cobalamin” OR “thiamine” OR “vitamin B1” OR “riboflavin” OR “vitamin B2”) AND (“diet” OR “supplementation” OR “nutrient intake” OR “serum levels”). We tailored this string according to the indexing systems of each database, using Boolean operators and truncations when appropriate.

**Figure 1:**
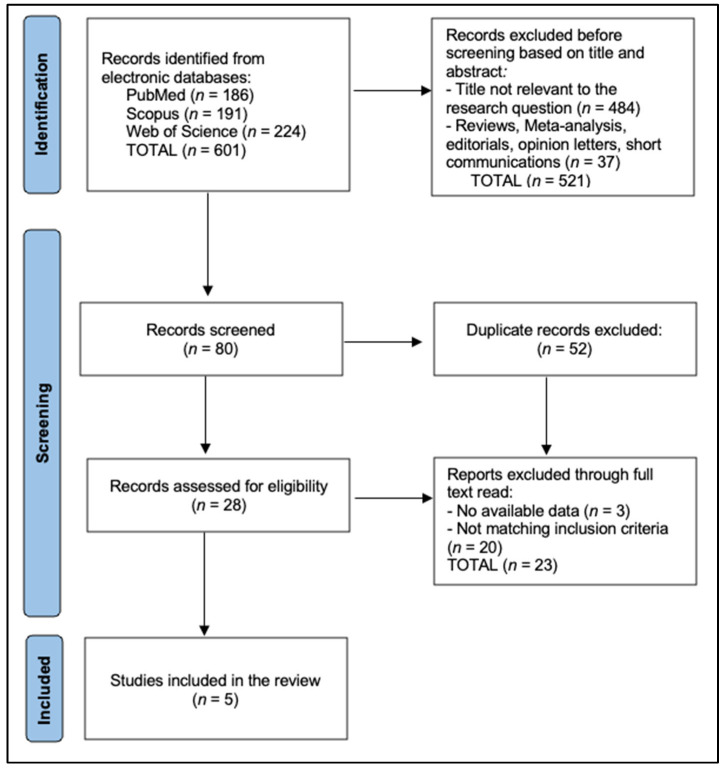
PRISMA flowchart.

We ran our search in MEDLINE (PubMed), Scopus, and Web of Science, limiting results to peer-reviewed human studies. To broaden coverage, we also used Google Scholar in a targeted fashion to identify gray literature, focusing on the first 200 results as recommended in search guidelines for broad-based searches. Next, we merged search outputs and removed duplicates using EndNote software v20 (Philadelphia, PA, USA). Two reviewers independently screened titles and abstracts for potential relevance, discarding studies that clearly did not meet inclusion criteria (e.g., animal or in vitro studies, articles not in English, or investigations that did not measure periodontal outcomes). For each abstract that passed this stage, we retrieved full texts for a second level of screening, as presented in Figure 1.

We applied our inclusion and exclusion criteria during full-text assessment. When key information was missing, we attempted to contact the corresponding authors to clarify study details. After finalizing the included articles, we performed backward and forward citation tracking on each eligible study to identify any additional references. This exhaustive approach ensured that key data linking the vitamin B complex to periodontal disease measures were captured.

### 2.3. Data Extraction and Quality Assessment

Data extraction was conducted in duplicate by two investigators (R.B. and B.A.B.) using a standardized form. The following information was extracted from each included study: (1) authors and publication year; (2) study design (e.g., cross-sectional, retrospective cohort); (3) sample size and demographic characteristics (age, sex distribution, and relevant health status); (4) method of vitamin B assessment, including whether intake was ascertained by dietary survey or supplementation records, or measured through serum biomarkers; (5) periodontal disease classification, such as clinical attachment level, probing depth, and other validated or self-reported periodontal outcomes; (6) confounding variables accounted for, such as age, smoking, and diabetes; and (7) main findings, including risk estimates, *p*-values, and effect sizes.

The 2018 World Workshop on the Classification of Periodontal and Peri-Implant Diseases introduces a comprehensive system to classify periodontitis into stages and grades [2]. Stage I denotes initial periodontitis with up to 2 mm of clinical attachment loss and predominantly horizontal bone loss. Stage II is characterized by moderate periodontitis with 3–4 mm of attachment loss and up to 33% horizontal bone loss. Stage III represents severe periodontitis, featuring over 4 mm of attachment loss, tooth mobility due to vertical bone loss exceeding 33%, and potential for further tooth loss. Stage IV indicates very severe periodontitis with extensive tooth loss and significant reduction in masticatory function due to advanced bone loss and periodontal destruction. The grades reflect the progression rate and risk factors: Grade A shows slow progression without additional risk factors; Grade B indicates a moderate progression possibly influenced by controllable risk factors like smoking fewer than 10 cigarettes per day or managed diabetes; and Grade C describes rapid progression, often exacerbated by significant risk factors such as smoking more than 10 cigarettes per day or uncontrolled diabetes. This detailed classification aids in the precise diagnosis and personalized management of periodontitis, considering both the severity of the disease and the patient’s overall health status.

To evaluate methodological quality and risk of bias, the Newcastle–Ottawa Scale (NOS) was employed for each observational study. This scale evaluates aspects such as the selection of study groups, comparability of groups, and ascertainment of exposure/outcomes. A maximum of nine stars can be awarded. Generally, studies with 7–9 stars are considered high quality, those with 4–6 stars are moderate quality, and those below 4 stars are low quality. Discrepancies between reviewers were resolved through discussion or adjudication by a third reviewer. We also scrutinized how potential confounders, including socioeconomic status, smoking, diabetes, and oral hygiene habits, were measured and adjusted in each study. Studies with robust statistical models and well-defined periodontal measurements were deemed stronger. While no formal scoring system was used to exclude studies, the NOS findings were used to interpret and weigh each study’s conclusions. Finally, the risk-of-bias assessment was summarized in a table.

### 2.4. Data Synthesis and Analysis

Following data extraction and quality assessment, we synthesized the findings narratively due to the heterogeneity in study designs, exposure measurements, and outcomes. Key characteristics and outcomes of each study were organized into summary tables for clarity. Because the data spanned both cross-sectional and retrospective cohort designs, a meta-analysis was deemed inappropriate. In addition, the parameters of vitamin B supplementation or dietary intake were inconsistent (ranging from direct serum folate measurements to self-reported intake frequencies), precluding direct quantitative pooling of effect sizes.

Instead, we performed a descriptive review focusing on each B vitamin’s association with periodontal outcomes. We coded associations as positive (protective effect), negative (higher risk), or neutral (no statistically significant correlation) based on each study’s reported results. Where available, we highlight effect measures such as odds ratios (ORs) or relative risks (RRs) for high vs. low exposure categories, while noting whether confounders (e.g., smoking status, age, and comorbidities) were adequately addressed.

Given the heterogeneity in study designs and outcome measures, a quantitative meta-analysis was not attempted. Instead, we employed a descriptive synthesis emphasizing consistency or divergence in vitamin B effects on periodontal indicators. We aggregated findings on folate, vitamin B12, B1, B2, and B6 separately, noting effect sizes (odds ratios, relative risks) where available.

## 3. Results

Table 1 outlines the fundamental characteristics of the five included studies, emphasizing differences in population demographics, study design, sample size, vitamin B complex focus, and method of periodontal disease assessment. Yu et al. [21] conducted a cross-sectional analysis in older adults using NHANES data from 2001 to 2002. They specifically investigated serum folate levels and correlated these with periodontal disease measurements defined by clinical attachment loss (CAL) and probing depth (PD). The sample size (n = 844) was comparatively modest, but the robust nature of the NHANES protocol, including standardized measurements, confers reliability to their findings.

In contrast, Hosoda et al. [22] narrowed their focus to young adult women (mean age ~20 years) in Japan, highlighting an underexplored demographic with emerging periodontal health challenges. Their sample was smaller (n = 120) but offered in-depth nutritional analysis using a diet history questionnaire (DHQ) and the community periodontal index (CPI). While the narrower age range enhances internal consistency, it limits generalizability to broader populations.

Luo et al. [23] took advantage of a large NHANES sample (n = 6415) to examine associations of B vitamins—such as B1, B2, B6, folate, and B12—measured by both dietary intake and self-reported supplementation with periodontal outcomes. Notably, they classified periodontal disease severity using both clinical measures (CAL, PD) and self-report, providing multiple perspectives on disease burden.

Meanwhile, Li et al. [24] expanded upon NHANES 2009–2014 data with an even larger sample (n = 8959), employing a dose–response analysis. This approach allowed them to identify not only whether abnormal B vitamin intake correlates with periodontal disease but also to investigate threshold or risk-point levels of these micronutrients. Finally, Watson et al. [25] leveraged the vast UK Biobank (n = 9476 included in their sub-analysis) to examine the link between nutrient intakes and self-reported periodontal disease risk, focusing on nutrient-based dietary patterns that included B vitamins. Their approach underscores the notion that total dietary patterns, rather than individual vitamins alone, might influence periodontal outcomes.

Across these studies, the design heterogeneity is notable: three cross-sectional analyses (Yu et al. [21], Hosoda et al. [22], Luo et al. [23]) plus one large cross-sectional from Li et al. [24] (NHANES subset) and one prospective approach in the UK Biobank (Watson et al. [25]). Each employed different measures of periodontal disease—ranging from full-mouth clinical metrics to self-reported painful/bleeding gums.

Table 2 summarizes participant demographics and key covariates each study incorporated into their statistical models. Yu et al. [21] focused on older Americans (mean age ~70.6 years), nearly half of whom were female. Prevalence of smoking and diabetes was notable, aligning with recognized risk factors for poor periodontal health. Their careful adjustment for confounders, such as vitamin B12 and homocysteine levels, underscores the complexity of nutrient interrelationships when examining folate’s role in periodontal status.

Hosoda et al. [22], in contrast, studied a cohort of young Japanese adult women (mean age 20.4). Neither smoking nor diabetes were reported in this group, likely reflecting the specific eligibility criteria. Instead, they accounted for chewing difficulty, body mass index (BMI), and dietary hardness—uncommon variables that provide interesting perspectives on how daily eating habits might tie into periodontal health.

Moving to the large NHANES-based analyses, Luo et al. [23] and Li et al. [24] each included participants with mean ages in the early-to-mid 50s, capturing a broader adult range. Smoking rates varied (26.7% in Luo et al. vs. 17.8% in Li et al.), and diabetes rates similarly ranged from 10.0% to 15.6%, demonstrating that cardiometabolic comorbidities are common confounders in large US populations. Both studies factor in multiple covariates—such as hypertension (HTN), BMI, and race/ethnicity—to isolate the vitamin B complex effects more accurately.

Finally, Watson et al. [25] recruited participants from the UK Biobank with a mean age of 56.2 years, predominantly female (57.2%) and older, with a relatively low smoking rate (6.6%). Instead of using a direct measure of social disadvantage such as the US-based poverty index ratio, they employed the Townsend Deprivation Index (TDI) as a proxy for socioeconomic status. Similar to the NHANES studies, Watson et al. adjusted for age, BMI, smoking status, and more, emphasizing the importance of confounder control.

Table 3 focuses on the specific vitamin B measurements—whether dietary intake or serum levels—and the operational definition of periodontal disease used across the studies. Yu et al. [21] measured serum folate in older adults, employing a radio protein binding assay kit. Their periodontal outcome definition combined a probing depth ≥ 5 mm and attachment loss ≥ 3 mm in at least one site, consistent with standard clinical thresholds. They reported that each standard deviation increase in folate levels was linked to approximately 26% lower odds of periodontitis, highlighting a strong protective effect for older individuals.

Hosoda et al. [22] used dietary intake questionnaires (DHQs) to gauge B vitamin consumption in young adult women while employing the community periodontal index (CPI) to classify periodontal condition. They found that inadequate vitamin B2 and B6 intake correlated with deeper pockets (i.e., CPI codes 3 or higher), suggesting that even in young populations with minimal confounders such as smoking, suboptimal B vitamin diets could predispose to early periodontal changes. By contrast, Luo et al. [23] integrated dietary intake data (including supplementation) from NHANES participants, linking B1, B2, B6, folate, and B12 with standardized periodontal metrics. Notably, they combined objective (CAL, PD) with subjective (self-report of PD severity) measures. Their key finding was that inadequate thiamine (B1), riboflavin (B2), or cobalamin (B12) significantly elevated the probability of moderate-to-severe periodontitis, whereas adequate riboflavin was protective.

Li et al. [24] similarly used NHANES data but adopted a dose–response approach to detect thresholds. They discovered that moderate (yet sufficient) levels of B vitamins, especially folate and B1, correlated with reduced periodontal disease severity, but beyond a certain intake, no additional protection occurred. This underscores a nuanced, possibly nonlinear, relationship. Lastly, Watson et al. [25] approached the issue by identifying “nutrient-based dietary patterns” in the UK Biobank. Their “high micronutrient” pattern, which included B6, folate, and other vitamins, was inversely associated with self-reported painful or bleeding gums. They estimated around a 20% risk reduction in the highest quartile of micronutrient intake. This approach diverges from analyzing single vitamins in isolation, hinting that synergy among B vitamins (and other nutrients) could have meaningful impacts on periodontal status.

Table 4 presents the statistical models and risk estimates central to understanding each study’s main conclusions. Yu et al. [21] used a multivariable logistic regression that adjusted for key demographic and biochemical factors. Their reported odds ratio (OR) of an approximately 0.74 per standard deviation increase in serum folate underscores the protective relationship. By also controlling for homocysteine levels, the study addresses an important mechanistic pathway, given that elevated homocysteine can exacerbate inflammatory conditions.

Hosoda et al. [22] implemented a multiple regression approach after a preliminary screening of variables. They discovered that lower B2 and B6 intakes significantly predicted higher CPI scores, independent of confounders such as BMI, chewing difficulty, and snack intake. This suggests that the link between B vitamins and periodontal health in young adults is not merely an artifact of body weight or snacking patterns. Luo et al. [23] employed ordinal regression, classifying periodontal disease into severity tiers (mild, moderate, severe). They found that insufficient thiamine (B1) predicted an elevated odds ratio of 1.33 for more severe periodontal disease. Notably, the presence of protective associations for B2 was also confirmed, with an OR around 0.90, reinforcing the notion that higher riboflavin intake confers benefit.

Li et al. [24] delved deeper into potential threshold effects using restricted cubic spline (RCS) modeling. Their dose–response analysis indicated a nonlinear association, especially for folate and vitamin B1. Up to a certain intake level, risk reduction was notable (~30% reduction), but additional intake beyond that threshold did not confer further protection. This approach highlights the complexity of micronutrient effects on periodontal status. Finally, Watson et al. [25] performed logistic regression on a “nutrient-based pattern” encompassing multiple vitamins, including B6 and folate. Their key finding was that those in the highest quartile of this nutrient-rich pattern had an OR of about 0.76 compared to the lowest quartile, suggesting a roughly 24% risk reduction in self-reported periodontal symptoms. Importantly, controlling for the Townsend Deprivation Index (TDI) and other lifestyle confounders underscores the pattern’s robustness.

## 4. Discussion

### 4.1. Summary of Evidence

The variability in the observed effects of vitamin B on periodontitis across studies can be attributed to differences in study design, population demographics, and the specific types of vitamin B examined. For example, larger studies with robust methodologies like those conducted by Luo et al. [23] and Li et al. [24], which utilized extensive NHANES data, consistently demonstrate stronger associations between vitamin B levels and periodontal health due to their comprehensive control over confounding factors such as age, smoking status, and socioeconomic background. In contrast, smaller or more homogeneous studies, such as the one by Hosoda et al. [22], focused on young Japanese women, and might not show as pronounced effects due to limited dietary variability and fewer confounders. Furthermore, the biochemical mechanisms linking vitamin B to periodontal health, notably its role in inflammation reduction and homocysteine metabolism, can vary significantly across populations. These mechanisms are crucial as vitamin B helps modulate inflammatory processes and maintain the structural integrity of the periodontal ligament and bone, key factors in the progression of periodontitis.

One noteworthy aspect is the breadth of populations, from older adults [21] to young women [22], to large cross-sectional national cohorts [23,24] and a British biobank sample [25]. Despite the differences in demographic profiles and measurement techniques, the converging message is that insufficient vitamin B, especially folate, correlates with exacerbated periodontal disease markers. Another promising finding is that some threshold or dose–response relationships may exist, as indicated by Li et al. [24] using restricted cubic spline analyses. Understanding that more is not always better helps refine dietary recommendations and underscores the importance of balanced, rather than excessively high, intake.

Mechanistically, B vitamins could mitigate periodontal disease by lowering systemic inflammation, improving collagen synthesis and epithelial turnover, and moderating homocysteine levels. For example, the well-documented link between folate deficiency and elevated homocysteine may provide a route by which poor folate status potentiates local inflammatory cascades. The interplay of folate with vitamins B12 and B6 in single-carbon metabolism further suggests that deficiencies in more than one B vitamin could exacerbate the same inflammatory or immunologic pathways. Meanwhile, the synergy of the B complex with other dietary factors is evident in the Watson et al. study [25], which identified a “high micronutrient” dietary pattern that collectively lowers periodontal risk.

In exploring the impact of B vitamins on oral health, the study conducted by Rodrigo F. Neiva et al. [26] revealed that supplementation with a vitamin B complex following access flap surgery (AFS) in patients with chronic periodontitis led to significant improvements in clinical attachment levels (CALs). Specifically, the vitamin B-supplemented group experienced a mean increase in CAL of +0.41 ± 0.12, contrasting sharply with the placebo group, which saw a decrease of −0.52 ± 0.23 (*p* = 0.024). Furthermore, this benefit was pronounced in both shallow and deep periodontal sites, with the supplemented group showing superior results in clinical parameters post-surgery. In a similar vein, a narrative review by Cristina-Crenguța Albu et al. [27] emphasized the essential role of folic acid (FA), another member of the vitamin B family, in maintaining oral health through its influence on cellular processes and genetic transcription. The review highlighted the impact of FA deficiency on periodontal health, pointing to increased oxidative stress and genomic instability as key factors in deteriorating periodontal conditions. Both studies underline the crucial role of B vitamins in oral health, demonstrating their potential to enhance periodontal healing and counteract the effects of deficiencies at the molecular level.

The studies by Shivayogi M. Hugar et al. [28] and Geng Zong et al. [29] both explored the relationship between vitamin B12 levels and various oral health outcomes, yet in different demographic and methodological contexts. Hugar et al.’s cross-sectional study [28] on children aged 10 to 14 revealed a high prevalence of vitamin B12 deficiency, found in 64% of participants, with deficient children exhibiting significantly higher decayed, missing, and filled permanent teeth (DMFT) scores and worse gingival indices compared to those with normal vitamin B12 levels. The Pearson’s correlation coefficients (DMFT: −0.614, PI: −0.663, GI: −0.477) indicated a strong inverse relationship between vitamin B12 levels and both caries and gingival disease severity. In a similar manner, the longitudinal study by Zong et al. [29] assessed adult participants over approximately six years and found that lower baseline vitamin B12 levels were associated with greater progression in periodontal disease and a higher risk of tooth loss. Those in the lowest quartile of vitamin B12 levels experienced more significant increases in probing pocket depth (PD) and clinical attachment loss (CAL), and had a 57% higher risk of tooth loss compared to those in the highest quartile. These studies collectively underscore the potential importance of vitamin B12 in maintaining oral health across different ages, with both indicating that lower vitamin B12 levels could contribute to the exacerbation of oral health issues.

The studies by Jung-Hoo Lee et al. [30] and Shima Ghasemi et al. [31] both examine the role of the vitamin B complex in relation to different dental outcomes, highlighting the potential and limitations of these vitamins in dental health and post-surgical recovery. Lee et al. [30] investigated the association between dietary intake of thiamine, riboflavin, and niacin (vitamins B1, B2, and B3) and periodontal health among Korean adults. They found that the inadequate intake of vitamin B3 was associated with a higher prevalence of periodontitis, particularly among females and middle-aged adults, indicating a significant odds increase of 1.25 times for developing periodontitis. This suggests a protective role of adequate vitamin B3 intake against periodontal disease. In a similar manner, Ghasemi et al. [31] conducted a clinical trial to assess the effect of vitamin B complex supplementation on pain and sensory issues following mandibular implant surgery. Their findings, however, showed no significant differences in pain intensity and paresthesia rates between the control and intervention groups over a three-month period, suggesting that vitamin B complex supplementation might not have a significant impact on these specific post-operative recovery outcomes. These studies collectively emphasize the potential specific benefits of vitamin B in periodontal health, while also noting limitations in other aspects of dental recovery and treatment efficacy.

Despite these findings, the relationship between vitamin B intake and periodontitis is influenced by various factors including dental hygiene, systemic health conditions like diabetes, and renal function, as well as age, gender, and educational status [32]. These factors affect the body’s ability to utilize vitamin B effectively in maintaining periodontal health. For instance, diabetes exacerbates periodontal disease by impairing immune responses and nutrient metabolism, thus affecting periodontal tissue integrity [33]. Additionally, differences in dietary knowledge and choices, influenced by educational levels, impact nutrient intake including crucial vitamins [34]. Recognizing these multifaceted influences is vital for effectively managing periodontitis through tailored nutritional and therapeutic approaches.

Nevertheless, other potential confounding factors should be taken into consideration. For example, the basal metabolic rate, along with renal function and inflammatory status, significantly influences the relationship between vitamin B intake and its serum concentration. Given that vitamin B is water soluble, it is rapidly utilized or excreted, unlike fat-soluble vitamins which are stored and released more slowly. Therefore, factors like metabolic rate could notably affect vitamin B’s bioavailability [35]. Additionally, renal function’s role in excreting excess vitamins and inflammation’s impact on metabolic processes could complicate this association. This highlights the importance of considering metabolic variations in nutritional assessments, especially for those at risk of vitamin B deficiency.

The systematic review highlighted substantial evidence supporting the hypothesis that adequate intake of the vitamin B complex is associated with improved periodontal outcomes. The findings across different populations and study designs consistently indicate that higher levels of certain B vitamins correlate with reduced odds of periodontal disease. For instance, in older adults, increased serum folate levels significantly lowered the odds of periodontal disease. Similarly, adequate intake of riboflavin and other B vitamins showed a protective effect against periodontitis in various cohorts. Conversely, insufficient intake of thiamine, riboflavin, and pyridoxine was associated with worse periodontal indices and increased disease likelihood. These results are consistent with the proposed immunomodulatory and tissue repair roles of vitamin B complex, suggesting a clear link between B vitamin status and periodontal health. Thus, the study hypothesis that adequate vitamin B intake or status is associated with better periodontal outcomes is convincingly confirmed by the reviewed evidence.

It is worth mentioning that the belief that the vitamin B complex’s effect on periodontal health might be overinterpreted is well founded, given the myriad of factors that can impact periodontal conditions. Indeed, while studies like those conducted by Yu et al. [21] and Luo et al. [23] report a correlation between vitamin B levels and reduced periodontitis, these effects are generally modest. The impact of vitamin B is nuanced and may be overshadowed by other significant determinants such as age, smoking, diabetes, and socioeconomic factors, all of which profoundly influence periodontal health. For instance, despite rigorous statistical adjustments in these studies, the intrinsic complexities of individual health profiles and lifestyle factors might dilute the perceivable benefits of vitamin B on periodontal outcomes. This suggests a need for cautious interpretation of vitamin B’s role against a backdrop of broader periodontal disease determinants.

Future studies should further explore the causal mechanisms through which vitamin B complex intake influences periodontal health. Prospective cohort studies or randomized controlled trials are needed to establish definitive causality and to evaluate the impact of specific vitamin B supplementation on periodontal disease progression. Additionally, exploring the synergistic effects of combined B vitamin complexes, rather than individual vitamins, on periodontal outcomes could provide insights into more effective nutritional interventions.

### 4.2. Limitations

Several limitations hamper the present review. First, the five included studies are predominantly cross-sectional in design, preventing the establishment of causality. Second, the measurement methods for vitamin B intake varied, with some relying on self-reported 24 h recalls or dietary frequency questionnaires and others using serum biomarkers; these discrepancies could influence the accuracy of exposure estimates. Third, the definitions and diagnostic criteria for periodontal disease are not fully uniform, spanning from self-reported loose teeth or bleeding gums to thorough clinical attachment level assessments. This heterogeneity complicates direct comparisons across studies. Fourth, many investigations adjusted for common confounders like smoking and BMI, but residual confounding from unmeasured factors (e.g., oral hygiene frequency, psychosocial stress, or genetic predispositions) cannot be ruled out. Moreover, self-reported periodontal disease is a major limitation, as it cannot be considered a clinical periodontal outcome. Another limitation is that only studies written in English were considered, as well as excluding gray literature. A significant limitation of this analysis is the inability to differentiate between studies focusing on the “intake of vitamin B” versus those examining “serum concentration of vitamin B”, due to the insufficient data available within the five studies included in our review. Finally, there is limited exploration of synergy among B vitamins themselves or with other micronutrients, an area that requires further study to optimize potential guidelines on the vitamin B complex and periodontal disease prevention.

## 5. Conclusions

In conclusion, emerging data indicate that inadequate intake or status of the vitamin B complex, particularly folate, is consistently associated with a greater risk or severity of periodontal disease. While the precise mechanisms await further elucidation, maintaining adequate B vitamin intake appears beneficial for periodontal health. Clinicians and public health policymakers may consider encouraging balanced diets rich in B vitamins—potentially through whole-food sources—to support oral health. Future prospective and interventional studies with standardized periodontal measurements will be necessary to substantiate causality and refine intake recommendations, thus capitalizing on the promise of vitamin B complex as an adjunct in periodontal disease management.

## Figures and Tables

**Table 1:** Characteristics of the included studies.

Study (Author, Year)	Country	Population	Study Design	Sample Size	Vitamin B(s) Studied	Periodontal Assessment	Risk-of-Bias Assessment (NOS Score, Key Notes)
Yu et al. (2007) [21]	United States	Older adults (≥60 years)	Cross-sectional (NHANES)	844	Folate (Serum Levels)	Clinical exam (CAL, PD)	High quality (8 stars); well-adjusted for confounders like age and smoking
Hosoda et al. (2021) [22]	Japan	Young adult women (~20 y)	Cross-sectional	120	B vitamins from dietary intake	CPI (Codes 0–4)	Moderate quality (5 stars); limited by smaller sample size and narrower demographic
Luo et al. (2024) [23]	China	Adults ≥ 30 years	Cross-sectional (NHANES)	6415	B1, B2, B6, folate, B12 intake	CAL, PD, self-report	High quality (9 stars); extensive sample and robust confounder adjustments
Li et al. (2022) [24]	United States	Adults (NHANES 2009–2014)	Cross-sectional	8959	B vitamins (intake, dose–response)	Clinical PD categories (mild–severe)	High quality (7 stars); comprehensive dose–response analysis
Watson et al. (2022) [25]	United Kingdom	Middle-aged/older adults	Cohort (UK Biobank)	9476	B6, B12, folate, others included	Self-reported PD risk (questionnaire)	High quality (8 stars); large cohort, diverse metrics

**Table 2:** Population demographics and key covariates.

Study	Mean Age (Years)	Female (%)	Smoking (%)	Diabetes (%)	BMI (Mean/Range)	Covariates Adjusted
Yu et al. [21]	70.6	49.6	12.6	16.8	27.0 (avg BF 5.2%)	Age, sex, race, BMI, vit B12, etc.
Hosoda et al. [22]	20.4	100	0	0	~20.3	Age, BMI, chewing difficulty, etc.
Luo et al. [23]	52.9	51.3	26.7	10	29.5	Age, sex, race, edentulism, etc.
Li et al. [24]	52.4	48.8	17.8	15.6	29.5	Age, gender, smoke, hypertension
Watson et al. [25]	56.2	57.2	6.6	NR	26.8	Age, diabetes, BMI, smoke, alcohol

NR—Not Reported; BMI—Body Mass Index.

**Table 3:** Vitamin B complex measurements and periodontal outcomes.

Study	Vitamin B Measurement	Outcome Definition	Key Vitamin B Findings
Yu et al. [21]	Serum folate (ng/mL) via RIA kit	PD: ≥5 mm at ≥1 site, CAL ≥3 mm at ≥1 site	Lower serum folate correlated with increased PD; each SD increase in folate reduced risk by ~26%
Hosoda et al. [22]	Dietary B intake from DHQ (mg/day)	CPI ≥ Code 3 for PD classification	Lower B vitamin (esp. B2, B6) associated with deeper pockets and higher CPI codes
Luo et al. [23]	Self-reported B1, B2, B6, folate, B12 intake	CAL, PD, and self-report PD severity (mild–severe)	Inadequate B1, B2, and B12 linked to higher PD risk; adequate B2 protective.
Li et al. [24]	24 h recall + supplement dose–response	PD categories: mild, moderate, severe	Demonstrated threshold effects for folate, B1, B2, and B12; excessive intake not beneficial.

PD—Probing Depth; CAL—clinical attachment level; RIA—Radioimmunoassay; SD—standard deviation; DHQ—diet history questionnaire; CPI—community periodontal index.

**Table 4:** Risk estimates and statistical outcomes.

Study	Main Statistical Model	Confounders Controlled	Risk Estimate (95% CI) for Key B Vitamin(s)
Yu et al. [21]	Multivariable logistic regression	Age, sex, race, BMI, B12, homocysteine	OR~0.74 (0.58–0.93) per SD increase in serum folate
Hosoda et al. [22]	Multiple regression after 2-step screening	BMI, chewing difficulty, snack intake	B2 *p* < 0.05, B6 *p* < 0.01 for higher CPI code
Luo et al. [23]	Ordinal regression for PD severity	Smoking, hypertension, diabetes	Inadequate B1 OR 1.33, *p* < 0.05; B2 protective (OR 0.90)
Li et al. [24]	RCS (restricted cubic spline) dose–response	Age, sex, race, smoking, BMI	Nonlinear thresholds for B1, folate; up to ~30% risk reduction
Watson et al. [25]	Logistic regression on “nutrient-based pattern”	Diabetes, BMI, smoking, alcohol	Q4 vs. Q1: OR ~0.76 (0.65–0.90) for high B6/folate pattern

BMI—Body Mass Index; OR—Odds Ratio; SD—standard deviation; CI—Confidence Interval; CPI—community periodontal index; PD—Periodontal Disease.
